# A Single Transradial Guiding Catheter in Patients With ST-Segment Elevation Myocardial Infarction Undergoing Percutaneous Coronary Intervention: A Door-to-Balloon Optimized Strategy

**DOI:** 10.7759/cureus.46802

**Published:** 2023-10-10

**Authors:** Jorge Antonio Cervantes-Nieto, Juan Andres Pimentel-Esparza, Omar Gomez-Monterrosas, Heidy Jaqueline Casillas-Gastelum, Juan Alan Fuentes Mendoza

**Affiliations:** 1 Cardiology, National Institute of Cardiology Ignacio Chávez, Mexico City, MEX; 2 Internal Medicine, Hospital Regional de Pemex en Salamanca, Salamanca, MEX; 3 Interventional Cardiology, Angeles Hospital Puebla, Puebla, MEX; 4 Internal Medicine, Xoco General Hospital, Mexico City, MEX

**Keywords:** ischemic heart diseases, transradial guiding catheter, door-to-balloon time, st-segment elevation myocardial infarction (stemi), percutaneous coronary intervention

## Abstract

Background

Acute coronary syndrome (ACS) is the leading cause of morbidity and mortality worldwide. The different reperfusion strategies have evolved over the years, and efforts have been directed to reduce its complications. Among these strategies, the one that has shown the best results is percutaneous coronary intervention, which has significantly improved the survival and prognosis of these patients; however, this procedure is not free of complications since multiple factors are involved. Among them is the time of patient care from the time of diagnosis until the coronary reperfusion therapy is performed.

Methodology

In this study, we describe the experience in our center with the 6-French Ikari Left guide catheter as a strategy of radial angiography-angioplasty with a single catheter to reduce the care times of patients with acute ST-elevation myocardial infarction (STEMI) in our center and compare it with the series reported by other international centers since. To establish an alternative to the usual approach that consists of the use of Judkins catheters, diagnosis, and guiding.

Results

Our study showed a success rate for diagnostic angiography and percutaneous coronary intervention (PCI) with the 6- French Ikari Left catheter comparable to those obtained in other centers, even with lower complication rates than the usual approach with Judkins’ Catheters.

Conclusions

The use of the 6-French Ikari Left catheter demonstrated shorter needle-device time and compared to other international series, it was shown to be shorter and related to shorter fluoroscopy time. Our study has a small sample and only included a highly selected population, which represents a limitation. This study is vulnerable to the different practices of the operators, with involvement in procedure time and use of contrast volume.

## Introduction

Ischemic heart disease is the leading cause of death in Mexico. The incidence of ST-elevation myocardial infarction (STEMI) ranges from 43 to 144 per 100,000 person-years. The hospital mortality rate for acute myocardial infarction in elderly patients (> 45 years) is three times higher in Mexico than the average of the Organization for Economic Co-operation and Development (OECD) countries, being 28.1 vs. 7.5 deaths per 100 discharges, respectively [1].

STEMI, according to the fourth universal definition, is ST-segment elevation in two contiguous electrocardiogram leads associated with symptoms of ischemia and elevation of markers of myocardial damage (defined as troponin elevation with at least a value above the 99th percentile of the upper reference limit) [2]. Treatment of STEMI is reperfusion therapy, either with percutaneous coronary intervention (PCI) or fibrinolysis, with primary angioplasty being the preferred strategy in patients presenting within the first 12 hours of the onset of clinical manifestations [3]. Zijlstra et al. demonstrated in 1993 the superiority of percutaneous transluminal coronary angioplasty (PTCA) vs. thrombolytic treatment in acute MI [4]. This was corroborated by Weaver et al. who evidenced a reduction in mortality, reinfarction, and cerebrovascular events with the mechanical intervention [5]. This benefit is derived from a high success rate in opening the artery responsible for the infarct by angioplasty (>90%), compared to 50-60% success with thrombolytics, which are also associated with an intracranial bleeding rate of 1-2% [4].

This treatment strategy reduces mortality, as Dalby et al. showed a 19% reduction in all-cause mortality when patients were transferred to centers with PTCA [6]. The door-to-balloon time (D2B) is a parameter both in the quality of medical care and as a predictor of 30-day mortality, as shown by Berger et al., who analyzed this outcome in their study comparing mortality of two groups divided into time intervals: in the first 60 minutes it was 1%, in 61-75 minutes it was 3.7%, in 76-90 minutes it was 4%, in more than 90 minutes it was 6.4%, and in those in which balloon angioplasty was not performed it was 14.1%, clearly showing the reduction in mortality at a lower D2B [7]. Due to the above, in the European Society of Cardiology clinical practice guidelines, a D2B of less than 90 minutes is recommended from diagnosis to transfer to centers with primary angioplasty [3].

In their study, Ikari et al. used a single catheter to perform diagnostic and therapeutic angiography. They reported a success rate of 97% in a series of 103 patients undergoing transradial coronary interventions, using 6 Fr Ikari guiding catheters [8]. On the other hand, Youssef et al. described, in 2008, the experience in their center of using a single catheter to diagnose and treat coronary lesions with a success of 96.8% was found to cannulate the radial artery [9]; in 98.1% of the cases, angiography of both coronary arteries was performed, and in 96.6% of the cases, it was possible to cannulate the two vessels with adequate support. Torii et al. used this catheter for primary PCI in patients with STEMI [10]. The primary endpoint of this study was the “time frame” (TF), and they found that in the Ikari catheter group, the TF was 15 ± 11 minutes, and in the conventional group, 25 ± 11 minutes, with a statistically significant difference (p = 0.001). In addition, it was found that the D2B was lower in the study group compared to the conventional group (55 ± 16 vs 63 ± 17; p=0.01). The limitation of this study is that it was single-center and retrospective.

Another problem primary PCI faces is its number of complications since it can cause morbidity and mortality events, which is more evident in elderly patients with multiple comorbidities [4], and the nature of the coronary disease is likely to present more of a challenge to successful treatment [11]. The frequency of complication rates from PCI increases with age, and those with unstable symptoms have worse outcomes [12]. The most common complication seen during coronary angiography is observed at the trans-femoral vascular access (bleeding and vascular access complications), appearing in up to 5% of procedures [13-15]. Puncture site closure devices have been developed that reduce bed rest time, improve patient comfort, and shorten hospital stay, but have not modified the risk of bleeding and have brought new complications such as infections, embolization, or arterial stenosis [16]. Although to a lesser extent, radial access can also present complications such as bruising or bleeding, which may require drainage or blood transfusion [17]. Other more serious but less frequent complications are the formation of pseudoaneurysms, arteriovenous fistulas, arterial thrombosis (1-4%), and ischemia of the hand [18]. Several types of guide catheters have been designed to achieve coronary angiography with less manipulation using a radial approach, a single catheter to perform right and left coronary angiography, and percutaneous coronary intervention, which reduces procedure times, fluoroscopy, costs, and complications such as radial spasm [9,19]

Within these multipurpose catheters, we find the Ikari curved guiding catheter (Terumo Corporation, Tokyo, Japan), which has two designs. The 6-Fr Ikari Left catheter is used in our center for right and left coronary intervention.

## Materials and methods

This was a prospective randomized study to know the impact of using the 6-Fr Ikari Left guide catheter on D2B, TF, and cardiovascular mortality. This is the first study conducted in Mexico as a strategy to reduce the D2B in a national reference center.

Patients admitted with a diagnosis of STEMI at the National Institute of Cardiology Ignacio Chávez, Mexico City, Mexico, between July 2018 to June 2019 were included in the study. Inclusion criteria were patients of all genders, over 18 years of age, who attended the emergency room in the first 12 hours after the onset of the STEMI, and who agreed to perform PCI. Patients who presented the following criteria were excluded: (i) Non-palpable radial pulse, (ii) Temporary pacemaker placement before PCI, (iii) Arteriovenous fistula in the right thoracic limb, (iv) Presence of ascending thoracic aortic aneurysm, (v) Patients who underwent an interventional procedure other than PTCA.

For quantitative variables, the mean and median were used as measures of central tendency, and standard deviation and minimum and maximum values as measures of dispersion, and for qualitative variables, the Chi-square statistical test was used. This study was approved by the National Institute of Cardiology Ignacio Chávez, Mexico (approval number: 0792762).

## Results

This study included 43 patients, of whom 35 (81.4%) were men and eight (18.6%) were women. The median age of the participants was 58 years. The most frequent comorbidities found in the participants were 22 cases of systemic arterial hypertension (51%), 16 of diabetes mellitus (37%), and 10 of dyslipidemia (23%). The rest of the baseline demographic variables are shown in Tables 1, 2.

**Table 1:** Demographic characteristics of patients PCI: percutaneous coronary intervention Data have been represented as n and %

Variable	Number	Percentage
Female	8	18.6
Male	35	81.4
Systemic arterial hypertension	22	51
Type 2 diabetes mellitus	16	37
Dyslipidemia	10	23
Smoking	25	58
Insulin use	2	4.6
Previous myocardial infarction	5	11.6
Previous revascularization surgery	1	2.3
Previous PCI	3	6.9
Hypothyroidism	2	4.6
Chronic Kidney Disease	1	2.3

**Table 2:** Additional demographic characteristics of patients BMI: body mass index; GFR: glomerular filtration rate; NT-pro-BNP: N-terminal fragment brain natriuretic peptides; KK: Killip & Kimball classification; TIMI: thrombolysis in myocardial infarction; STEMI: ST-segment elevation myocardial infarction; GRACE: Global Registry of Acute Coronary Events; LDL: low-density lipoprotein; HDL: high-density lipoprotein Data have been represented as Mean±SD

Variable	Mean value ± SD
Age (years)	58 ± 8.9
BMI	26.55 ± 3.2
Total cholesterol (mg/dl)	161 ± 41
LDL cholesterol (mg/dl)	98 ± 37
HDL cholesterol (mg/dl)	36 ± 11
Triglycerides (mg/dl)	183 ± 122
GFR (ml/min/1.73m^2^)	91 ± 25
NT-pro-BNP (pg/ml)	2125 ± 3027
Troponin I (ng/ml)	18.23 ± 26.5
KK	1 ± 1.3
TIMI STEMI Score	2.7 ± 2.0
GRACE Score	123 ± 35

A subgroup analysis of the angiographic and electrocardiographic findings was analyzed. Most of the infarcts are in the inferior segments, with the right coronary artery as the main culprit artery in 60% (n=26) and 46% (n=20) of the cases, respectively, followed by anterior infarcts caused by occlusion of the left anterior descending artery in 32% (n=14) and 30% (n=13) of the cases. The rest of the information about the analysis is presented in Table 3.

**Table 3:** Angiographic and electrocardiographic characteristics LAD: left anterior descending artery; Cx: circumflex artery; RCA: right coronary artery; LCA: left coronary artery Data have been represented as n and %

Variable	Number	Percentage
Myocardial infarction location		
Anterior	14	32
Lateral	1	2.3
Inferior	26	60.4
Posterolateral	2	4.65
Related coronary artery		
LAD	13	30
Cx	9	20
RCA	20	46
LCA	1	2.3

Continuing with the analysis related to the interventional procedure and its related complications, it was seen that the average delay time was 218 minutes; this time translates as the time from the onset of symptoms until the patient was admitted to our Institute. D2B was 72 minutes, and the needle-to-device time, the time the radial puncture was performed until the guidewire crossed the infarction plaque, was an average of 17 minutes. It should be noted that this time includes all the procedures until the guidewire crosses the infarction plaque, rather than the diagnostic coronary angiography of the right and left coronary systems. Most patients presented a 0 thrombolysis in MI (TIMI) flow in the culprit artery before the procedure with a mean post-intervention TIMI flow of 2.76; an average of 1.25 stents per procedure were placed using the delayed stent technique in two of the cases (4.6%) and placement of stents; the mean diameter and length of the stents were 3.35 mm and 25.3 mm, respectively. Direct stenting was performed in seven cases (16.27%); thrombus aspiration was performed only in one patient (2.3%). Among the complications the patients presented, the most frequent was radial vasospasm appearing in four cases (9%), followed by heart failure, contrast nephropathy, ventricular fibrillation, and atrioventricular (AV) block, each of them appearing in two cases respectively (4.6%). No patient had a re-infarction during their hospital stay. The rest of the information about the analysis is presented in Tables 4, 5.

**Table 4:** Characteristics during the procedure (standard deviation) SD: standard deviation; D2M: door-to-balloon time; TF: time frame; TIMI: thrombolysis in myocardial infarction; PTCA: percutaneous transluminal coronary angioplasty; TMP: TIMI myocardial perfusion Data have been represented as Mean±SD

Variable	Mean value ± SD
Delay time (minutes)	218 ± 153
D2B (minutes)	72 ± 23
TF (minutes)	17 ± 29
Contrast volume (ml)	135 ± 57
Fluoroscopy time (minutes)	16.93 ± 11
Hospital stays (days)	5.1 ± 17
TIMI Pre-PTCA	0.3 ± 0.7
TIMI Post-PTCA	2.76 ± 0.4
TMP	2.69 ± 0.5
Number of stents	1.25 ± 0.6
Stent diameter	3.35 ± 0.5
Stent length	25.3 ± 6.2

**Table 5:** Eventualities present during the procedure VT: ventricular tachycardia; VF: ventricular fibrillation; AV: atrioventricular Data have been represented as n and %

Variable	Number	Percentage
Radial vasospasm	4	9
Vascular complications	0	0
Deferred stent	2	4.6
Direct stent	7	16.27
Aspiration thrombus	1	2.3
Deaths	2	4.6
Reinfarction	0	0
Recurring angina	1	2.3
Heart failure exacerbation	2	4.6
Contrast-induced nephropathy	2	4.6
VT/VF	2	4.6
AV block	2	4.6
Gastrointestinal bleeding	1	2.3

**Figure 1:**
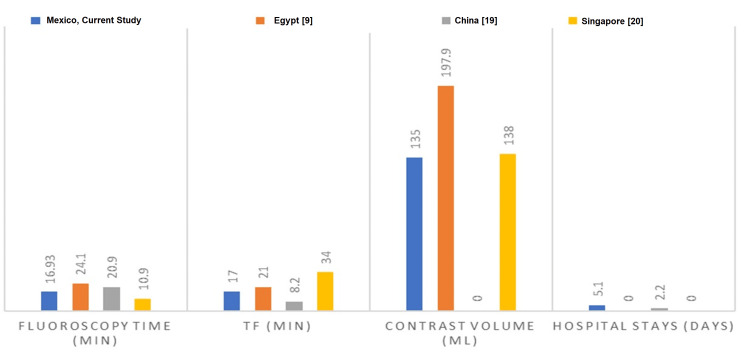
Characteristics during the procedure TF: time frame; Min: minute References: [9,19,20]

**Figure 2:**
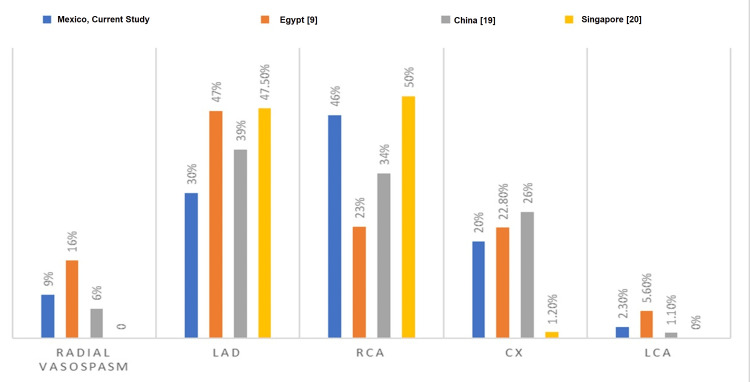
Related coronary artery LAD: left anterior descending artery; Cx: circumflex artery; RCA: right coronary artery; LCA: left coronary artery References: [9,19,20]

A sub-analysis of the procedures performed by physicians affiliated with the Interventional Cardiology Fellowship Program was performed to assess the ease of use of the Ikari catheter. The average number of procedures performed by first-year physicians in interventional cardiology fellowship was 4.59 ± 2.34, in second-year physicians, it was 3.94 ± 2.22, and for physicians attached to the Interventional Cardiology Service, it was 4.4 ± 1.67. A comparison of the outcomes of this study with other international studies is given in Table 6 and Figures 1-2.

**Table 6:** Comparison of the most important outcomes between the current study and studies from Egypt, China, and Singapore LAD: left anterior descending artery; Cx: circumflex artery; RCA: right coronary artery; LCA: left coronary artery Data have been represented as Mean±SD and %

Variables	Mexico (Current Study)	Egypt [9]	China [19]	Singapore [20]
Catheter	Ikari 3.5 6 Fr	Ikari 3.5 6 Fr	Terumo 5 Fr	Ikari 3.5 6 Fr
Myocardial infarction	100%	7.1%	6%	100%
Fluoroscopy time (minute), Mean±SD	16.93 ± 11	24.1 ±36.1	20.9±9.3	10.9
Time frame, Mean±SD	17 ±29	21.4±15.1	8.2 ±2.4	34
Contrast volume (ml), Mean±SD	135 ±57	197.9 ±46.2	-----	138
Hospital stays (days), Mean±SD	5.1 ±17	----	2.2±.5	-----
Radial vasospasm	9%	16%	6%	-----
LAD	30%	47%	39%	47.5%
RCA	46%	23%	34%	50%
Cx	20%	22.8%	26%	1.2%
LCA	2.3%	5.6%	1.1%	0%

## Discussion

The 6- Fr Ikari Left catheter multipurpose catheter demonstrated a reduction in D2B and needle-device time compared to other international series; this represents an alternative to improve care times in acute MI. Another finding of our study is the possible feasibility of using the 6-Fr Ikari Left guide catheter to perform right and left coronary angiography and treat occluded arteries in the context of STEMI. The use of this catheter offers some advantages, such as decreased procedure time and less exposure to radiation due to less fluoroscopy time and less use of contrast; it also reduces pain at the radial artery and wrist at the time of catheter crossover.

Among the results observed in our study, it is noteworthy that both arteries were cannulated in all cases; using another catheter was not required due to the anatomy of the Ikari catheter that allows easy cannulation of both coronary sinuses. When comparing our study with the international series, we can see that fluoroscopy time was within the average of other centers and was even better than in the series from China, Egypt, and Singapore [19,9,20]. The Chinese study used a multipurpose catheter other than the Ikari, and the volume of contrast used by us was remarkably lower than that used by the Egyptian study. However, their study was not in the context of ACS; they were facing more complex scenarios such as chronic total occlusive lesions. Only one study reported days of in-hospital stay, and once again, only 6% of their procedures were in the context of acute MI, so it is logical that since most of the procedures are scheduled, they can even be managed on an outpatient basis [19]. In the current study, 100% of the participants were infarcted patients, patients who require more observation and complementary studies and may present more complications. With regard to complications, radial spasm was reported in two studies [9,19]; the number of radial spasms in the current study is below the mean of these two studies, which is 11.

With regard to the arteries responsible for the infarction and the arteries treated, it is noteworthy that in the study that focused only on STEMI [20], the right coronary artery was the culprit in most infarcts, followed by the anterior descending artery, reversing this relationship in the other two studies [9,19].

Keeping in mind all the above, we can say that the Ikari catheter is valuable in the context of ACS, giving satisfactory results in our center, which were comparable and even in some cases better than in other international series reported, and should be considered an excellent option to perform diagnostic coronary angiography and percutaneous trans coronary angioplasty.

The current study had a small sample size and only included a highly selected population, which represents a limitation. This study is vulnerable to the different practices of the operators, with involvement in procedure time and use of contrast volume. However, patients catheterized by trainee operators and experienced operators were also included, noting that despite the operator's experience, the catheter is friendly to successfully perform angiography and PCI.

## Conclusions

The use of the Ikari guide catheter as a single catheter to perform PCI in patients with acute MI with ST-segment elevation has proven to be a safe technique that helps reduce treatment times for these patients. Therefore, it should be considered as a technique of choice to optimize the interventional treatment of patients with STEMI. It has been demonstrated that it is a safe procedure and has low rates of failure or periprocedural complications, even when performed by personnel in training (Interventional Cardiology Fellows in training). Therefore, it is proposed to consider the use of single diagnostic-therapeutic catheters as strategies to optimize care times in patients with STEMI, to seek to reduce total ischemia times. Once this method is implemented, the impact of optimizing total ischemia times on mortality and major adverse cardiovascular events (MACE) must be studied to objectively evaluate the use of this versus the standard strategy and its impact on MACE.
